# LEA (Late Embryogenesis Abundant) proteins and their encoding genes in *Arabidopsis thaliana*

**DOI:** 10.1186/1471-2164-9-118

**Published:** 2008-03-04

**Authors:** Michaela Hundertmark, Dirk K Hincha

**Affiliations:** 1 Max-Planck-Institut für Molekulare Pflanzenphysiologie, Am Mühlenberg 1, D-14476 Potsdam, Germany

## Abstract

**Background:**

LEA (late embryogenesis abundant) proteins have first been described about 25 years ago as accumulating late in plant seed development. They were later found in vegetative plant tissues following environmental stress and also in desiccation tolerant bacteria and invertebrates. Although they are widely assumed to play crucial roles in cellular dehydration tolerance, their physiological and biochemical functions are largely unknown.

**Results:**

We present a genome-wide analysis of LEA proteins and their encoding genes in *Arabidopsis thaliana*. We identified 51 LEA protein encoding genes in the Arabidopsis genome that could be classified into nine distinct groups. Expression studies were performed on all genes at different developmental stages, in different plant organs and under different stress and hormone treatments using quantitative RT-PCR. We found evidence of expression for all 51 genes. There was only little overlap between genes expressed in vegetative tissues and in seeds and expression levels were generally higher in seeds. Most genes encoding LEA proteins had abscisic acid response (ABRE) and/or low temperature response (LTRE) elements in their promoters and many genes containing the respective promoter elements were induced by abscisic acid, cold or drought. We also found that 33% of all Arabidopsis LEA protein encoding genes are arranged in tandem repeats and that 43% are part of homeologous pairs. The majority of LEA proteins were predicted to be highly hydrophilic and natively unstructured, but some were predicted to be folded.

**Conclusion:**

The analyses indicate a wide range of sequence diversity, intracellular localizations, and expression patterns. The high fraction of retained duplicate genes and the inferred functional diversification indicate that they confer an evolutionary advantage for an organism under varying stressful environmental conditions. This comprehensive analysis will be an important starting point for future efforts to elucidate the functional role of these enigmatic proteins.

## Background

Late embryogenesis abundant proteins (LEA proteins) were first found in cotton (*Gossypium hirsutum*) seeds, accumulating late in embryogenesis [1]. They were subsequently found in the seeds of many other plants, but also in vegetative organs, especially under stress conditions such as cold, drought, or high salinity (see [2,3] for reviews). According to the appearance of different sequence motifs/patterns or biased amino acid composition, plant LEA proteins have been separated into different groups [4-7]. However, the grouping of proteins and the nomenclature of the groups have not been consistent in the literature (see [8] for a recent review).

LEA proteins are not plant specific. They have also been found in other organisms, such as the bacteria *Deinococcus radiodurans *[9] and *Bacillus subtilis *[10], the chironomid *Polypedilum vanderplanki *[11], the brine shrimp *Artemia *[12], different species of nematodes [13-15], rotifers [16,17] and cyanobacteria [18]. The presence of LEA proteins has been associated with cellular tolerance to dehydration, which may be induced by freezing, saline conditions, or drying. In extreme cases, organisms can even survive a complete loss of water (anhydrobiosis; see [19] for review). Sugars, especially the disaccharides sucrose and trehalose, are thought to play important roles in cellular desiccation tolerance [19], but it is clear that additional substances are necessary for a cell to attain anhydrobiosis [20,21]. Desiccation-tolerant rotifers can even survive complete desiccation without accumulating sugars [22], but they show enhanced expression of genes encoding LEA proteins during drying [16,17]. Likewise, a strong induction of *LEA *gene expression has been found in the desiccation tolerant resurrection plant *Craterostigma plantagineum *during slow drying [23]. These and many other examples in the literature suggest that LEA proteins may indeed be important determinants of cellular dehydration tolerance in a variety of organisms from bacteria to plants and lower animals.

A common feature of LEA proteins is a biased amino acid composition that leads to high hydrophilicity [24] and heat stability in solution. This is similar to the recently developed concept of "hydrophilins" [25] and indeed many LEA proteins were classified as hydrophilins by these authors. However, since a distinguishing feature of hydrophilins is a high glycine content, not all LEA proteins were classified as hydrophilins and instead other non-LEA proteins were included. The functional significance of membership in either or both of these groups is unclear. The resolution of this and many other questions concerning LEA proteins is severely hampered by the fact that, although these proteins have been known for 25 years, only limited functional information is available.

The overexpression of genes encoding LEA proteins can improve the stress tolerance of transgenic plants. Expression of the barley gene *HVA1 *in wheat and rice conferred increased drought tolerance to plants [26,27] and expression of the wheat genes *PMA80 *and *PMA1959 *increased the dehydration tolerance of transgenic rice [28]. The cold tolerance of transgenic tobacco was increased by the expression of a citrus gene encoding a LEA protein (CuCOR19; [29]). Likewise, the freezing tolerance of Arabidopsis was increased by the ectopic expression of the wheat gene *WCS19 *[30], the Arabidopsis gene *COR15A *[31], and the co-expression of the genes *RAB18 *and *COR47*, and *XERO2 *and *ERD10 *[32]. The freezing tolerance of strawberry leaves was enhanced by expression of the wheat dehydrin gene *WCOR410 *[33]. Mutant analysis showed that the EM6 protein is required for normal seed development in Arabidopsis [34]. On the other hand, the expression of two cold-induced LEA proteins from spinach [35] and three desiccation-induced LEA proteins from *C. plantagineum *[36] in tobacco did not induce any significant changes in the freezing or drought tolerance of the respective transgenic plants. This may indicate either that not all LEA proteins make a significant contribution to plant stress tolerance, or that they need a particular background to function in, as suggested for transgenic strawberry plants [33].

An alternative approach for *in vivo *functional studies is the expression of LEA proteins in yeast or bacteria. Such studies have shown that a wheat LEA protein conferred tolerance against hyperosmotic stress to *Saccharomyces cerevisiae *cells [37], while LEA proteins from *Chlorella*, tomato and barley protected yeast cells against high salt concentrations and freezing [38-40]. Likewise, a LEA protein from soybean increased the salt tolerance, but not the tolerance against hyperosmotic stress, when expressed in *Escherichia coli *[41].

Parallel efforts have concentrated on determining biochemical and biophysical activities of these proteins. A stabilization of lactate dehydrogenase and malate dehydrogenase during freezing and/or drying has been shown for LEA proteins from citrus [42,43], *Chlorella *[44], barley [45], Arabidopsis, and *C. plantagineum *[46,47]. Fumarase and rhodanese could be stabilized during drying by the addition of a pea seed LEA protein [48], catalase by a citrus LEA protein [42], and citrate synthase by LEA proteins from wheat, the nematode *Aphelenchus avenae *[49] and the rotifer *Adineta ricciae *[16]. These data indicate that several LEA proteins have the ability to stabilize labile enzymes under stress conditions. However, since no systematic studies, including negative results, across different groups of LEA proteins have been reported, it can not be judged whether this is a general property of LEA proteins or whether specific structural requirements exist.

Only a few papers have investigated other functional properties of LEA proteins. The Arabidopsis dehydrin ERD10 binds more water during drying than non-LEA control proteins [50,51] and this and other dehydrins bind calcium, iron and other divalent cations in a phosphorylation-dependent manner [52-54]. Radical scavenging by a citrus LEA protein [29] and the stabilization of dry sugar glasses by LEA proteins from *Typha latifolia *[55] and soybean [56] have also been reported.

These data indicate that LEA proteins have interesting functional properties related to their presumed role as cellular stabilizers under stress conditions. Unfortunately, the available data are too fragmented between species, structural groups, and methodologies to draw any general conclusions about structure-function relationships and physiological roles of LEA proteins. Such knowledge is not only of great basic scientific interest, but would also help to lead transgenic approaches and the technical use of LEA proteins as biostabilizers beyond mere trial and error. To obtain such knowledge, systematic biochemical, functional and physiological studies are required. Before such studies can be undertaken, genome-wide approaches are necessary to describe and classify the entire LEA complement of model organisms. We present such an analysis of LEA proteins and their respective genes in *Arabidopsis thaliana*. We correct previous annotation errors and annotate new genes, resulting in the identification of 51 genes in Arabidopsis that encode LEA proteins. Gene expression data, together with *in silico *analyses of promoter elements, and of the structure, localization and biochemical properties provide a comprehensive view of this enigmatic group of proteins.

## Results and Discussion

### LEA protein encoding genes in the Arabidopsis genome

Existing annotation and BLAST searches of well-characterized *LEA *genes from cotton (*Gossypium hirsutum*) identified 64 genes in the Arabidopsis genome that encode LEA proteins. To characterize and classify the genes, Pfam family domains were searched in the protein sequences (Table 1). Previously, LEA proteins have been separated into different groups [4-7], but the classification varies between different authors. For a better overview and tracking of proteins, we use the Pfam nomenclature, as this is uniquely related to sequence motifs. To allow easy reference to LEA proteins described in earlier publications, Table 2 compares the Pfam nomenclature with the two most frequently used systems proposed by Dure [5,6] and Bray [4].

**Table 1:** Characteristics of genes encoding LEA proteins in *Arabidopsis thaliana*

**Number**	**AGI code**	**Description NCBI**	**Pfam family**	**group**	**GRAVY**	**predicted subcellular localization**	**expression**
**1**	At1g01470	LEA14	LEA_2	LEA_2	0.056	other	Everywhere
**2**	At1g02820	LEA3 family protein	LEA_3	LEA_3	-0.491	Chloroplast	Stress
**3**	At1g03120	seed maturation family protein *	SMP	SMP	-0.564	other	Seed
**4**	At1g20440	Dehydrin, COR47	dehydrin	dehydrin	-1.257	other	Non-seed + stress
**5**	At1g20450	dehydrin ERD10, LTI45	dehydrin	dehydrin	-1.34	other	Non-seed + stress
**6**	At1g32560	group 1 domain-containing protein	LEA_1	LEA_1	-1.042	other	Seed
**7**	At1g52690	similar to LEA protein from *B. napus*	LEA_4	LEA_4	-1.317	other	Bud, seed + stress
**8**	At1g54410	dehydrin family protein	dehydrin	dehydrin	-1.868	other	Non-seed + stress
**9**	At1g72100	LEA domain-containing protein	LEA_4	LEA_4	-0.46	secreted	Seed
**10**	At1g76180	dehydrin ERD14	dehydrin	dehydrin	-1.265	other	Non-seed
**11**	At2g03740	LEA domain-containing protein	LEA_4	LEA_4	-0.703	Chloroplast	Bud
**12**	At2g03850	LEA domain-containing protein	LEA_4	LEA_4	-0.496	Chloroplast	Bud
**13**	At2g18340	LEA domain-containing protein	LEA_4	LEA_4	-0.93	secreted	Seed
**14**	At2g21490	dehydrin family protein	dehydrin	dehydrin	-1.032	other	Seed
**15**	At2g23110	similar to LEA proteins	-	PvLEA18	-1.059	other	Seed
**16**	At2g23120	unknown protein	-	PvLEA18	-1.001	other	Everywhere
**17**	At2g33690	similar to PvLEA18	-	PvLEA18	-1.311	other	Bud
**18**	At2g35300	LEA_1 domain containing protein	LEA_1	LEA_1	-1.156	other	Salt
**19**	At2g36640	LEA protein AtECP63	LEA_4	LEA_4	-1.023	other	Seed
**20**	At2g40170	Em-like protein GEA6/EM6	LEA_5	LEA_5	-1.407	other	Everywhere
**21**	At2g41260	Late embryogenesis abundant protein M17	-	AtM	-0.704	secreted	Seed
**22**	At2g41280	Late embryogenesis abundant protein M10	-	AtM	-0.011	secreted	Seed
**23**	At2g42530	cold-regulated protein COR15b	-	LEA_4	-0.542	Chloroplast	Non-seed + stress
**24**	At2g42540	cold-regulated protein COR15a	-	LEA_4	-0.554	Chloroplast	Non-seed + stress
**25**	At2g42560	LEA domain-containing protein	LEA_4	LEA_4	-0.978	other	Seed + salt
**26**	At2g44060	LEA domain-containing protein	LEA_2	LEA_2	-0.314	other	Non-seed + stress
**27**	At2g46140	LEA domain-containing protein	LEA_2	LEA_2	0.123	other	Seed + root
**28**	At3g02480	ABA-responsive protein-related	LEA_4	LEA_4	-1.213	other	Reproductive, seed + salt
**29**	At3g15670	LEA domain-containing protein	LEA_4	LEA_4	-1.369	other	Seed
**30**	At3g17520	LEA domain-containing protein	LEA_4	LEA_4	-1.047	secreted	Seed
**31**	At3g22490	RAB28	SMP	SMP	-0.193	other	Seed
**32**	At3g22500	Seed maturation protein AtECP31	SMP	SMP	-0.341	other	salt
**33**	At3g50970	dehydrin Xero2/LTI30	dehydrin	dehydrin	-1.173	other	Everywhere
**34**	At3g50980	dehydrin Xero1	dehydrin	dehydrin	-1.053	other	Seed
**35**	At3g51810	putative embryonic abundant protein AtEM1	LEA_5	LEA_5	-1.468	other	Seed
**36**	At3g53040	LEA domain-containing protein	LEA_4	LEA_4	-1.194	other	Seed
**37**	At3g53770	LEA protein-related	LEA_3	LEA_3	-0.79	Mitochondrion	Seed
**38**	At4g02380	LEA_3 family protein SAG21	LEA_3	LEA_3	-0.36	Chloroplast	Everywhere
**39**	At4g13230	LEA domain-containing protein	LEA_4	LEA_4	-0.831	Mitochondrion	Bud
**40**	At4g13560	LEA domain-containing protein	LEA_4	LEA_4	-1.181	other	Reproductive
**41**	At4g15910	drought-responsive protein AtDI21	LEA_3	LEA_3	-0.526	Chloroplast	Everywhere
**42**	At4g21020	LEA domain-containing protein	LEA_4	LEA_4	-1.291	Mitochondrion	Seed
**43**	At4g36600	LEA domain-containing protein	LEA_4	LEA_4	-1.072	Mitochondrion	Seed
**44**	At4g38410	putative dehydrin	dehydrin	dehydrin	-1.629	other	Root
**45**	At4g39130	dehydrin family protein	dehydrin	dehydrin	-0.774	other	Seed + bud
**46**	At5g06760	LEA group 1 domain-containing protein	LEA_1	LEA_1	-0.815	other	Seed + salt
**47**	At5g27980	seed maturation family protein	SMP	SMP	-0.373	other	Bud
**48**	At5g44310	LEA domain-containing protein	LEA_4	LEA_4	-1.409	Chloroplast	Seed
**49**	At5g53260	seed maturation family protein	SMP	SMP	-0.273	Chloroplast	Seed
**50**	At5g53270	seed maturation family protein	SMP	SMP	-0.103	other	Seed
**51**	At5g66400	dehydrin RAB18	dehydrin	dehydrin	-1.182	other	Seed + stress

The annotation and description in the NCBI database, and the protein family domains according to the Pfam database. The numbers in the first column are used throughout the paper as a shortcut to unambigously identify the different genes and proteins. GRAVY (grand average of hydropathy) quantitates the hydrophilicity of the proteins based on amino acid composition. Subcellular localization was predicted from protein sequence analysis using the targetP algorithm. The expression information is based on the quantitative RT-PCR experiments reported in Figure 4, Table 4 and 5 and Additional file 3.

**Table 2:** The nomenclature of the different LEA protein groups in the Pfam database and according to Bray [4] and Dure [6].

**Pfam**	**Bray**	**Dure**
dehydrin	group 2	D-11
LEA_1	group 4	D-113
LEA_2		LEA14; D-95
LEA_3		LEA5; D-73
LEA_4	group 3; group 5	D-7; D-29
LEA_5	group 1	D-19
SMP	group 6	D-34

The applied Pfam gathering threshold ensured that reliable results were retrieved from matching Pfam domains to the queried protein sequences. Thirteen genes were removed from the set of 64 (Additional file 1) because they had no significant LEA Pfam domain. It is striking that three of the removed genes contain a "root cap" Pfam domain. They were annotated as related to a LEA protein from *Picea glauca*, the EMB7 protein, which occurs late in embryogenesis. This LEA protein carries a root cap family domain, which, however, is not a signature domain of LEA proteins. Of the 13 Arabidopsis genes that were erroneously annotated (Additional file 1), 12 show similarities to *Picea glauca *genes which are expressed late in embryogenesis and therefore named *LEA *despite the fact that they have different structural domains.

**Figure 1:**
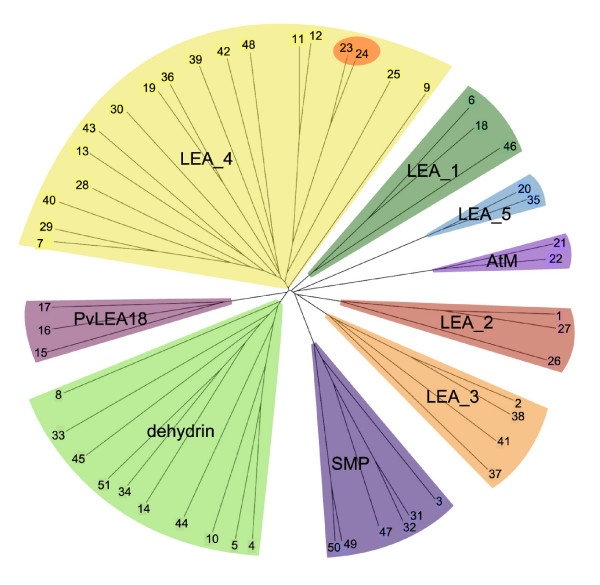
**Unrooted dendrogram of all Arabidopsis *LEA *genes**. Sequence alignments were performed unsing the ClustalW algorithm and an unrooted dendrogram was drawn subsequently. The different LEA groups are indicated by different colors, *COR15A *and *COR15B *are highlighted in the LEA_4 group.

We have classified two proteins (COR15A and COR15B) into LEA_4 that had previously been annotated as LEA proteins, although they do not contain a characteristic Pfam domain (Table 1, #23 and #24) above the Pfam gathering threshold. The two encoding genes form a tandem repeat and while COR15B contains a LEA_4 Pfam domain with a significant p-value of 0.046, the alignment for COR15A is not significant. However, we chose to include both genes in our list and in the following studies because they are structurally and functionally closely related and cluster together with other LEA_4 proteins (Fig. 1). We also included two novel LEA groups in our studies that do not have Pfam entries yet, the two AtM genes [57] and three genes homologous to the LEA18 gene from *Phaseolus vulgaris *[58]. These groups were included in our studies because of the similarity to known LEA proteins, namely high hydrophilicity, high expression levels during late embryogenesis and/or under abiotic stress conditions and lack of homology with other protein families. This led to the final annotation of 51 genes in the Arabidopsis genome that encode LEA proteins and these are listed in Table 1 with a numbering according to their position in the Arabidopsis genome, starting at the top of chromosome 1. This simplified numbering is used in the remainder of the paper to identify the corresponding genes and proteins. In the TIGR5 *Arabidopsis thaliana *database, nine of these 51 genes were not annotated as LEA, dehydrin or seed maturation protein, while seven were annotated as LEA but lacked significant Pfam domains and had high similarity to non-LEA protein families (Additional file 1).

**Figure 2:**
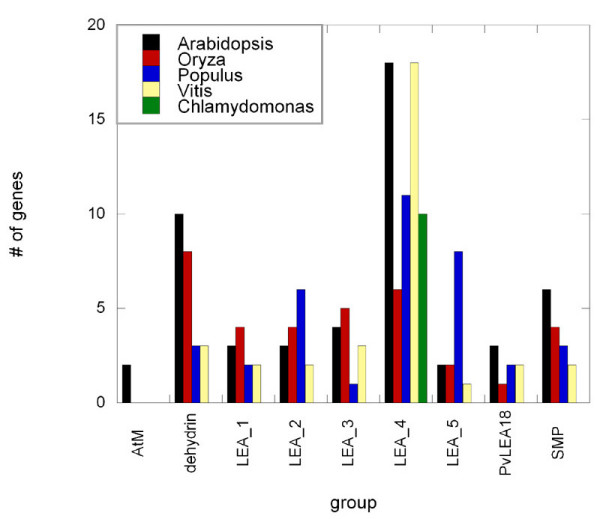
Comparison of the sizes of the different *LEA *gene groups in Arabidopsis, rice (Oryza), poplar (Populus), grapevine (Vitis) and Chlamydomonas.

To see whether such a large number of genes encoding LEA proteins is specific to Arabidopsis, we also searched the well-annotated rice genome using the same strategy as outlined above. In addition, the draft genomic sequences of grapevine (*Vitis vinifera*), poplar (*Populus trichocarpa*) und *Chlamydomonas reinhardtii *are also available and we extended our search to these species as well. We applied BLAST searches (expect-value cutoff 1e-5) with the *LEA *genes from *Gossipium hirsutum *and Arabidopsis to identify matching sequences. Since in these cases the BLAST search only returns positions on the scaffolds without any gene model information, this data should be considered as preliminary. The analyses revealed the presence of 35 *LEA *genes in rice, 36 in grapevine, 33 in poplar and only ten in Chlamydomonas, where only regions homologous to LEA_4 genes could be detected (Fig. 2). This may indicate that all other LEA groups evolved later in higher plants. This is consistent with the finding that the only *LEA *genes that can be detected in lower animals belong to the LEA_4 group [8]. In the rice genome, all genes have been previously annotated as encoding LEA, dehydrin or seed maturation proteins by the TIGR Community and an approach similar to ours identified 34 *LEA *genes in the rice genome [59]. If the number of genes in the different groups is compared between the investigated species (Fig. 2), the main differences occur in the dehydrin, LEA_4 and LEA_5 groups. The abundance of LEA_4 genes is lowest in rice, while especially Arabidopsis and grapevine have a large LEA_4 group. On the other hand, Arabidopsis and rice have about three times as many dehydrin genes as poplar and grapevine, but poplar has many more LEA_5 genes than all other species. There are also minor variations in the other groups, but except for the AtM there is at least one member of each group found in the investigated higher plant genomes. Interestingly, a BLAST search found the AtM genes to occur only in Brassicaceae species. Whether these differences between species have any functional significance is currently unknown and awaits functional characterization of the proteins.

### Characteristics of the encoded LEA proteins

We performed a ClustalW alignment of all 51 LEA proteins in Arabidopsis and the resulting unrooted dendrogram shows that the identified LEA groups are quite distinct from each other (Fig. 1). This result is not unexpected since the historical annotation as LEA is due to the expression pattern and to sequence homology within groups, but not between groups.

For a better overview of the characteristic features of the different LEA groups in Arabidopsis, we have compiled group-specific characteristics in Table 3. In the Arabidopsis genome, LEA_4 group (also known as group 3 or D-7) is the most dominant containing 18 members. This group is very heterogeneous and the gene products differ greatly in size and GRAVY (Grand Average of Hydropathy) index. They also lack high sequence similarity (data not shown) and no determinant motif could be found by the PRATT algorithm. In the majority of the protein sequences, the classical proposed motif (TAQAAKEKAXE; [6]) could not be found. Although the LEA proteins in *G. hirsutum *show this conserved motif, it seems to be quite variable in LEA_4 group genes from other species. However, the LEA_4 group proteins contain the characteristic Pfam domain which we used as the determinant criterion. We also found LEA_4 domains in homologues of the D-29 proteins that make up LEA group 5 [4,6]. Because the LEA_4 domain was present in proteins from both groups, we combined these groups under their Pfam name (Table 2).

**Table 3:** Group-specific values for the different calculated traits

**group**	**# of genes**	**GRAVY**			**Molecular weight**			**localization**	**motifs**
		**min.**	**max.**	**median**	**min.**	**max.**	**median**		

AtM	2	-0.704	-0.011	-0.358	11432	29559	20496	secreted	
dehydrin	10	-1.868	-0.774	-1.220	10796	29928	18881	other	[KR]-[1]-K-[DE]-K-[1]-P-G
									S(5)-[DE]-x-[DE]-[GV]-x(1,4)-[GE]-x(0,1)-[KR](4)
LEA_1	3	-0.815	-1.156	-1.042	10481	16179	13850	other	
LEA_2	3	-0.314	0.123	-0.045	16563	36036	17846	other	G-L-x(2)-[2]-[AILV]-x-[IV]-x-[GV]-x(2)-[PT]-x-[PS]-[ILV]-[NPST]-x(2)-[GI]
LEA_3	4	-0.790	-0.360	-0.509	9298	14418	10959	chloroplast and mitochondrion	W-x(2)-D-P-x-T-G-x-[WY]-x-P-x-[DGNST]
LEA_4	18	-1.409	-0.460	-1.035	7145	67195	26804	in all cellular compartments	-
LEA_5	2	-1.468	-1.407	-1.438	9934	16612	13273	other	G-[EQ]-T-V-V-P-G-G-T
PvLEA18	3	-1.311	-1.001	-1.059	7515	9713	8482	other	E-D-Y-K-x(2)-[AG]-Y-G-[AT]-[EQRS]-G-H
SMP	6	-0.564	-0.103	-0.307	16661	26826	19229	mostly other	-

The range and the median values for the GRAVY and molecular weight. The cellular localization based on the prediction with targetP represents the majority of the group proteins. The motifs specific for the groups were partly taken from Prosite (for dehydrins), the other motifs were build with the PRATT tool on the basis of well-defined LEA proteins belonging to these groups.

**Figure 3:**
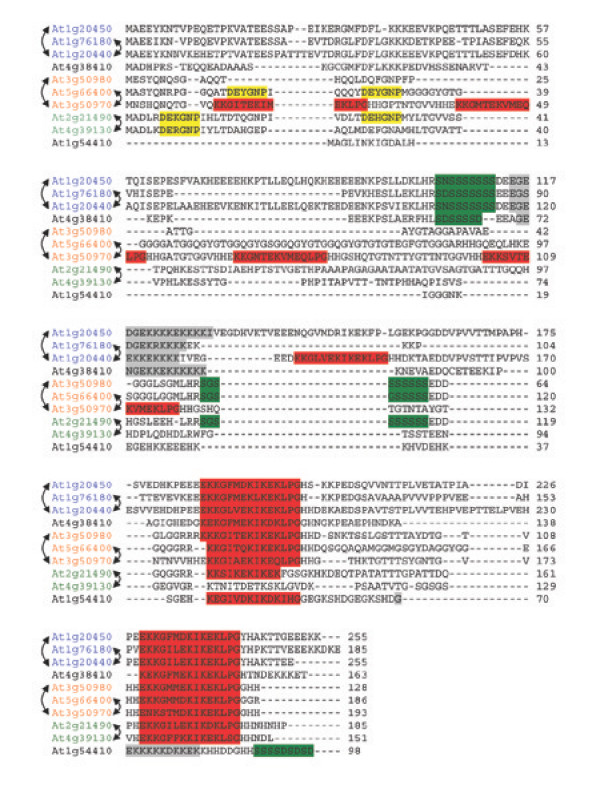
**Alignment of the dehydrin protein sequences of *Arabidopsis thaliana***. Amino acid sequences were aligned using the ClustalW algorithm. Dashes indicate gaps introduced for optimal alignment. The typical dehydrin sequence elements are highlighted: K segment – red; Y segment – yellow; S segment – green; Lys-rich segment – grey. The genes forming homeologous pairs and tandem repeats in the genome (compare Fig. 6, Table 6 and 7) are indicated by arrows on the right and left side of the gene identifier, respectively. The complete sequences can also be found in Additional file 5.

The second biggest group is the dehydrin group (also called group 2 or D-11) which includes ten genes. This is similar to the number found in rice (eight (Fig. 2); [59]) and in barley (13; [60]), but much more than in poplar and grapevine (Fig. 2). The Arabidopsis dehydrins show high sequence similarity at least in some parts and two common motifs are available in the Prosite database [5,61,62]. They have also been subdivided into an acidic group containing COR47 (#4), ERD10 (#5), ERD14 (#10), #14, #44, and #45, and a basic/neutral group containing #8, XERO2 (#33), XERO1 (#34), and RAB18 (#51) [53]. Figure 3 shows an alignment of all Arabidopsis dehydrin sequences. The characteristic K, Y, S, and Lys-rich segments are highlighted [63]. The KYS classification for all dehydrin proteins is given in Additional file 2. It can be seen from Figure 3 that only the K segment is present at least once in all dehydrins, making it the distinguishing feature of this group. The S segment, on the other hand, is present in eight, the Lys-rich segment in five, and the Y segment only in three of the ten proteins. Interestingly, the Lys-rich segment is only present in those LEA proteins that were highly expressed exclusively in vegetative tissues. The presence of any of the other segments was not indicative of one of the three possible expression patterns (seed, non-seed, seed+non-seed; compare Table 1).

Only the functional significance of the S-segment is known. It is phosphorylated, leading to calcium binding activity in some, but not all, investigated dehydrins [53]. The role of phosphorylation in those proteins that do not bind calcium is unclear, as is the physiological significance of the calcium binding activity.

The SMP group (seed maturation protein, D-34 or group 6; Table 2) has six members, while the remaining groups consist only of two to four members. Apart from the original six groups, we included some unusual groups in our study. The LEA_3 group (D-95 or LEA5; Table 2) is also characterized by a Pfam entry. Along with this group, the LEA_2 genes (LEA14 or D-74; Table 2) have been identified in cotton [64]. They encode 'atypical' LEA proteins because of their more hydrophobic character. The three PvLEA18 proteins belong to a small family of hydrophilic proteins that are related to a LEA protein in *Phaseolus vulgaris *that was reported to be induced upon dehydration [58]. The two AtM LEA proteins [57] are also hydrophilic and are expressed late in embryo development.

### Expression analysis of all Arabidopsis genes encoding LEA proteins

**Figure 4:**
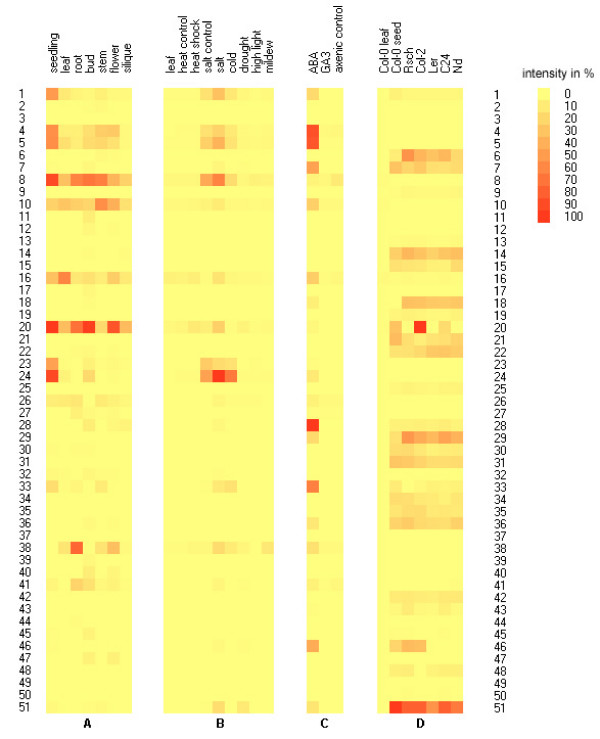
**Expression analysis of all 51 *LEA *genes in *A. thaliana***. Expression was measured by quantitative RT-PCR in different organs **(A)**, in mature leaves under different stress conditions **(B)**, in axenic cultures under hormone induction **(C) **and in mature seeds **(D)**. The color coding represents relative gene expression from 0 (yellow) to 100% (red), with 100% representing the highest expression within a given panel (compare e.g. the same leaf data as represented in A and D). See Additional file 3 for the complete data set. The numbers on the sides refer to the different *LEA *genes that are listed in Table 1.

For the 51 *LEA *genes identified in the Arabidopsis genome, expression analysis was performed on samples from different organs and in leaves under various, mainly abiotic, stress conditions. A detailed compilation of all *LEA *gene expression data is provided in Additional file 3. Figure 4A shows the expression of the genes in various organs, with the exception of seeds, which are shown in Figure 4D. In total, 22 of the 51 genes (43%) showed high expression levels (relative expression >10) in the non-seed organs in the absence of a stress or hormone treatment. Due to the high sensitivity of the Real-Time PCR measurements, expression at lower levels was detectable for all genes at least in some non-seed tissues. Transcript levels for most of these genes were highest in seedlings. The expression of *LEA *genes in green siliques was low compared to the other organ samples, indicating that the onset of the expression of seed-specific *LEA *genes had not yet been reached. It has been shown before that LEA transcripts accumulate immediately before maturation drying and remain stable in the desiccated seeds [1,65].

Of the 22 genes highly expressed in non-seed tissues, 12 were induced more than 3-fold by different stresses (the transcripts for #10 and #26 were induced less than 3-fold; the transcripts for #11, #28, #40, #45 and #47 were undetectable in leaves and the transcripts #12, #17 and #27 were lowly expressed in leaves under control and all stress conditions; Additional file 3). The expression of *LEA *genes was enhanced mainly by cold, drought and salt treatment (Fig. 4B, Table 4). Under cold conditions, besides the well-known cold-regulated genes [3]*COR47 *(#4), *ERD10 *(or *LTI45*, #5) *COR15A *(#24), *COR15B *(#23), and *XERO2 *(#33), several other genes were induced (#1, #38, #41, #46, #51). Under salinity stress, the expression differed in some cases from the expression under cold stress. In addition to the genes #33 (*XERO2*), #38 (*SAG21*), #41 (*AtDI21*) and #51 (*RAB18*) that were upregulated under both conditions, the genes #7, #16 and #20 (*EM6*) were salt-induced. *COR15A *(#24) and *COR15B *(#23) were highly upregulated under cold stress (more than 80-fold), whereas salinity and drought stress enhanced the expression about 2-fold (*COR15A*) or even decreased the expression (*COR15B*). Cold induction had been described for these genes before [66]. Drought treatment only enhanced the expression of four genes, #7, #41 (*AtDI21*), #46 and #51 (*RAB18*), in our experiments. The induction of the genes under drought conditions seems to be high (up to 55-fold), however, the expression levels of the genes are very low in unstressed leaves and still low in drought-stressed leaves compared to other stress treatments. Expression of *RAB18 *(#51) was enhanced more strongly under drought stress than under cold and high salinity conditions in accordance with earlier reports [67]. High light treatment had only small effects on *LEA *gene expression, with five genes (#7, #23, #24, #41 and #51) induced over 3-fold. The upregulation of *LEA *gene expression under high light conditions has been described previously [68,69]. Infection with powdery mildew enhanced the expression of eight genes, however, mostly to a smaller extent than abiotic stress, while heat shock treatment only increased the expression of *RAB18 *(#51) more than 3-fold.

**Table 4:** Stress induced expression of *LEA *genes. Highly expressed *LEA *genes that are induced at least 3-fold in stress-treated leaf tissue. The numbering is according to Table 1. The relative expression in the different samples is shown under Expression. The first three columns show the unstressed controls ("hydroponics" for salt stress, "heat control" for heat stress, "leaf" for the other conditions). The gene expression after stress treatment compared to the appropriate controls is shown under Induction. Bold-face numbers highlight induction of genes by more than 3-fold.

		**EXPRESSION**							**INDUCTION**						
**No.**	**leaf**	**hydroponics**	**heat control**	**cold**	**drought**	**high light**	**salt**	**heat**	**mildew**	**cold**	**drought**	**high light**	**salt**	**heat**	**mildew**
1	32.1	426.0	32.1	340.7	90.5	53.5	875.1	44.6	81.9	**10.6**	2.8	1.7	2.1	1.4	2.5
4	22.8	427.9	34.4	313.4	34.2	51.6	619.4	41.3	60.5	**13.8**	1.5	2.3	1.4	1.2	2.7
5	47.4	675.9	34.4	392.3	83.0	82.2	1085.2	50.3	91.7	**8.3**	1.8	1.7	1.6	1.5	1.9
7	0.2	0.5	0.1	0.3	4.2	0.9	142.7	0.3	1.3	2.0	**27.3**	**6.0**	**261.8**	2.5	**8.3**
8	90.9	1236.8	111.3	496.3	106.6	185.1	1769.2	113.2	172.8	**5.5**	1.2	2.0	1.4	1.0	1.9
16	182.1	33.2	166.4	104.7	53.8	78.0	385.1	218.5	49.9	0.6	0.3	0.4	**11.6**	1.3	0.3
20	97.9	210.2	129.7	51.5	46.1	50.5	681.2	295.1	72.1	0.5	0.5	0.5	**3.2**	2.3	0.7
23	4.9	724.9	9.5	411.2	2.1	30.6	500.0	14.4	22.7	**83.4**	0.4	**6.2**	0.7	1.5	**4.6**
24	15.9	1345.2	37.7	1912.1	39.1	57.4	2862.4	31.0	53.7	**120.3**	2.5	**3.6**	2.1	0.8	**3.4**
33	3.7	31.0	11.2	411.1	9.7	2.2	295.8	5.9	22.0	**111.4**	2.6	0.6	**9.5**	0.5	**6.0**
38	37.1	97.8	54.7	268.8	35.0	22.4	496.3	103.2	343.0	**7.2**	0.9	0.6	**5.1**	1.9	**9.3**
41	0.9	4.8	0.4	18.8	13.0	28.5	126.1	1.0	16.0	**21.3**	**14.7**	**32.2**	**26.1**	2.6	**18.1**
46	0.4	0.0	0.3	1.9	18.8	0.7	58.4	0.7	2.0	**4.6**	**45.1**	1.7	**3635.6**	2.7	**4.8**
51	1.3	51.9	0.7	17.3	70.0	4.8	480.4	2.7	11.4	**13.6**	**55.1**	**3.8**	**9.3**	**3.9**	**9.0**

*SAG *(senescence-associated gene) *21 *(#38) is induced during natural [70] and ozone-induced senescence [71] and under drought stress [70]. Here we show its additional induction under cold and salt stress conditions. This is in agreement with the earlier hypothesis that *SAG21 *is not directly involved in senescence, but is rather a marker for the stresses associated with senescence and cellular degradation [70]. Among the 14 genes that were induced more than 3-fold under any stress condition (Table 4), five belong to the dehydrin group. Also, three LEA_4 genes, two LEA_3 genes, one LEA_2 gene, one gene each of the PvLEA18, the LEA_1 and the LEA_5 group were stress induced, while no members of the SMP and the AtM groups were induced under any of the tested stress conditions in leaves.

Treatment of axenic cultures with abscisic acid (ABA) resulted in high expression of 27 genes, whereas only 12 genes were highly expressed in soil-grown seedlings and 10 genes in the untreated axenic control (Fig. 4C, Table 5). Treatment with gibberellic acid (GA3) resulted in high expression of 10 genes, similar to the untreated control (Additional file 3), indicating that GA3 is not a major regulator of *LEA *gene expression. Of the 27 genes highly expressed in the ABA-treated cultures, 21 were induced more than 3-fold (Table 5). At least one member of each group, except for SMP and AtM was induced by ABA-treatment. The induction of most genes was high compared to the very low levels of expression in the untreated plants. For many of the induced genes, ABA induction has been previously reported, such as *COR47 *(#4), *ERD10 *(#5), *ERD14 *(#10), *COR15A *and *COR15B *(#24 and #23), *XERO2/LTI30 *(#33) and *RAB18 *(#51) [66,67,72]. We also found that *EM6 *(#20), one of the two members of the LEA_5 group, was expressed in non-seed organs and could be induced by ABA-treatment, but the homologous gene *EM1 *(#35), which was seed-specific, was not induced by ABA. Comparison of gene expression under stress conditions and ABA treatment showed the expected substantial overlap (compare Fig. 4A and 4C).

**Table 5:** ABA-induced *LEA *gene expression

	**EXPRESSION**		**INDUCTION**
**No.**	**axenic control**	**ABA**	**ABA**
1	12.94	319.63	**24.7**
4	57.38	1538.84	**26.8**
5	19.47	1476.52	**75.8**
6	0.28	12.02	**43.1**
7	0.07	817.98	**11911.0**
8	181.55	93.35	0.5
10	41.46	407.82	**9.8**
13	0.02	11.07	**663.4**
15	0.17	11.99	**69.1**
16	58.20	433.25	**7.4**
18	0.25	129.79	**509.3**
20	0	56.48	
23	0.71	21.49	**30.2**
24	0.19	190.69	**1001.6**
25	0.01	13.44	**937.2**
26	39.10	113.81	2.9
27	24.90	25.34	1.0
28	1.59	1739.22	**1092.1**
29	0.20	320.85	**1595.0**
33	9.30	1107.58	**119.1**
36	0.06	195.57	**3312.2**
38	40.78	249.63	**6.1**
41	53.15	117.89	2.2
42	0.06	17.46	**285.3**
43	0.10	22.63	**216.2**
46	0.31	726.86	**2374.7**
51	0	213.74	

Expression and induction of genes by abscisic acid treatment. The numbering of the genes is according to Table 1. Bold-face numbers highlight induction of more than 3-fold.

The expression pattern of *LEA *genes in seeds was drastically different from the pattern in all other tissues (Fig. 4D). Only ten genes were found to be highly expressed in both seeds and in non-seed tissues under any conditions (*LEA14 *(#1), #7, #16, *EM6 *(#20), #27, #28, *XERO2 *(#33), *SAG21 *(#38), *AtDI21 *(#41), and #45). The overall level of expression of *LEA *genes in seeds was much higher compared to the expression in vegetative tissues. In addition, more *LEA *genes (33; 65% of all *LEA *genes) were highly expressed in seeds than in non-seed organs (22; 43%). We also investigated *LEA *gene expression in seeds of five additional Arabidopsis accessions (Landsberg *erecta*, C24, Niederzens, Rschew, Columbia-2). The content of *LEA *transcripts was similar in all accessions (Fig. 4D, Additional file 3), but some striking differences (e.g. #6, #20) were also detected. It is unclear whether these differences have any influence on seed desiccation tolerance or longevity. Comparison of the expression of *LEA *genes after ABA treatment of vegetative plants and in seeds showed only a limited overlap (compare Fig. 4C and 4D), indicating different signal transduction pathways in the different tissues.

We detected transcripts of every gene in at least one sample (Additional file 3). We compared our expression data with the AtGenExpress Affymetrix array data [73] and found a significant correlation (p = 1.456e-44, R = 0.5578) between the data sets. This correlation strongly confirms the reliability of our measurements, considering the different growth conditions that were used to generate the two data sets. In addition, our experiments provide expression data on three *LEA *genes (#3, #49, #50) that are not represented on the Affymetrix ATH1 array.

### ABRE and LTRE *cis*-acting regulatory elements in the promoters of Arabidopsis genes encoding LEA proteins

Genes encoding LEA proteins are highly expressed during abiotic stress and in seeds (Fig. 4). The ABRE (ABA responsive element; [74]) plays a key role in ABA signalling during seed development and under abiotic stresses (see [75,76] for recent reviews), while the second prominent *cis*-element in relation to the expression of stress regulated genes in general and *LEA *genes in particular is the DRE/CRT/LTRE (drought responsive/C-repeat/low temperature response) element, which binds the CBF/DREB1 transcription factors (see [3,76] for reviews). We queried the PLACE database [77] for these elements in the -2000 nt promoter sequences of the genes and compared the occurrence in the *LEA *gene promoters with the occurrence in all promoters in the genome using the Fisher exact statistical test. This analysis showed that the ABRE core motif was overrepresented in the *LEA *gene promoters with a p-value of 2.7E-04 and the LTRE core motif with a p-value of 3.7E-05.

Closer analysis showed that 82% of all *LEA *genes contain the ABRE core motif in their -2000 nt promotor regions (compared to 58% of total genes in Arabidopsis), while 69% (compared to 40% of total genes) contain the LTRE core motif (Additional file 4). The majority of *LEA *genes (32 out of 42) containing an ABRE motif were highly inducible (> 3-fold) by ABA, but only 12 out of the 35 genes that have an LTRE element in their -2000 nt region were highly inducible by either cold or drought in our experiments. Conversely, only three genes that were highly ABA inducible did not contain an ABRE motif, while only one gene that was highly cold induced contained no LTRE motif. This indicates the importance of the CBF/DREB1 signal transduction pathway for the cold and drought regulation of *LEA *genes in the vegetative tissues of Arabidopsis. However, only three of the cold or drought induced genes were not induced by ABA, indicating also a possible substantial crosstalk between these signal transduction pathways.

Interestingly, there were only two *LEA *gene promoters that contained neither an ABRE nor an LTRE motif (*SAG21 *and #39). Of these genes, only *SAG21 *(#38) was strongly upregulated by ABA, salt and cold treatment and by powdery mildew infection (Table 4). *SAG21 *may therefore be an interesting candidate as a reporter gene for the detection of novel stress and ABA-regulated signal transduction pathways.

### Structure and subcellular localization of LEA proteins

The LEA groups show differences in structural features of their members. The mean values for the molecular mass of the proteins show that, in general, LEA proteins are relatively small, with most falling in a range from 10 to 30 kDa (Table 3). There are a few very small LEA proteins (<10 kDa), especially the members of the PvLEA18 group. In addition, also some larger (~65 kDa) LEA proteins can be found in the LEA_4 group. The most striking differences can be seen in the GRAVY values (Table 3), with the LEA_2 group the most hydrophobic and LEA_5 the most hydrophilic. The larger dehydrin and LEA_4 groups show a wide range of GRAVY values but are altogether quite hydrophilic. Characteristics and sequences of all proteins are given in Additional file 5.

The secondary structures of 17 plant LEA proteins, one LEA protein from a nematode and two from a rotifer have been experimentally determined. Four of these proteins belong to LEA_5 group [78-81]. They were all shown to be in random coil conformation in solution. For two of these proteins, partial structuring in the presence of trifluoroethanol (TFE) or during drying has been reported [79,80].

The dehydrin group is the best investigated group with eight analyzed proteins. Four of those come from *Arabidopsis thaliana *(COR47 (#5), LTI29/LTI45 (#6), LTI30/XERO2 (#35), RAB18 (#53); [82]). The others were from soybean (*Glycine max*; [83]), maize (*Zea mays*; [84]), cowpea (*Vigna unguiculata*; [85], and a resurrection plant (*Craterostigma plantagineum*; [86]). All showed random coil structure in solution.

Secondary structure content of LEA_4 group proteins was determined by Fourier-transform infrared spectroscopy (FTIR) for the D-7 protein from *Typha latifolia *[55], the GmPM16 protein from soybean [56], the LEAM protein from pea [87], and the Aav-LEA1 protein from the nematode *Aphelenchus avenae *[88]. FTIR enables measurements with proteins in solution and in the dry state. Since the proteins are present in desiccation tolerant tissues (*T. latifolia *pollen, soybean and pea seeds, and dry, viable nematodes) their response to desiccation is of particular interest. All three proteins have a random coil structure in solution, but adopt a largely α-helical structure during drying. Surprisingly, it was recently shown by circular dichroism (CD) spectroscopy that of two highly similar LEA_4 proteins from a rotifer, one (ArLEA1A) showed random coil structure in the hydrated state and high α-helix content after drying, while the other (ArLEA1B) was largely α-helical (84–87%) in both the hydrated and dry state [16]. We conclude from these data that LEA_4 group proteins have properties that allow them to adopt an α-helical structure and that this structure may be related to their cellular function in desiccation tolerance. Interestingly, these structural properties and cellular functions seem to be conserved between plants and animals.

The only protein from the SMP group to have its secondary structure determined is MtPM25 from *M. truncatula *[80]. It showed increased content of α-helices and β-sheets during drying. The only LEA protein demonstrated to have a defined secondary and tertiary structure in solution is the LEA_2 group protein LEA14 (#1) from Arabidopsis [89]. It contains one α-helix and seven antiparallel β-strands, as shown by x-ray diffraction on the crystallized protein. Currently, there is no structural information available for the other groups of LEA proteins.

The structural data indicate that most LEA proteins are "natively unfolded" or "intrinsically unstructured" in solution. Such proteins have been recognized in all investigated organisms, both by computational prediction and by experimental determination of secondary structure (see [90] for a recent review). In general, a combination of low hydrophobicity and large net charge are characteristic features of these proteins [91]. It has been estimated that as many as 30% of all proteins are either completely or partially disordered [92].

This indicates that unstructured proteins play important roles in cells, despite their apparent lack of a defined three-dimensional structure. Most of these proteins, however, are not completely devoid of structure, but contain residual, flexible structural elements [93], such as polyproline II (P_II_) helices [94]. It has been reported that such structural flexibility enhances the ability of proteins to bind to interaction partners, such as DNA, RNA, or other proteins [93,95,96]. Binding is often accompanied by a folding transition. It has been argued that such a "fly-casting" mechanism [97] has a much higher efficiency in the search for binding partners than the simple diffusion of a compact, folded protein. Potential binding partners of LEA proteins, however, remain to be identified, but could include other proteins, nucleic acids, or membranes that might be stabilized under stress conditions through such interactions.

In general, the available data on the secondary structure of LEA proteins do not allow conclusions on the structural characteristics of the different groups, because too few members have been investigated, and they were not all investigated under the same set of conditions. Therefore, more systematic structural and functional characterization will be necessary to define structure-function relationships in LEA proteins.

**Figure 5:**
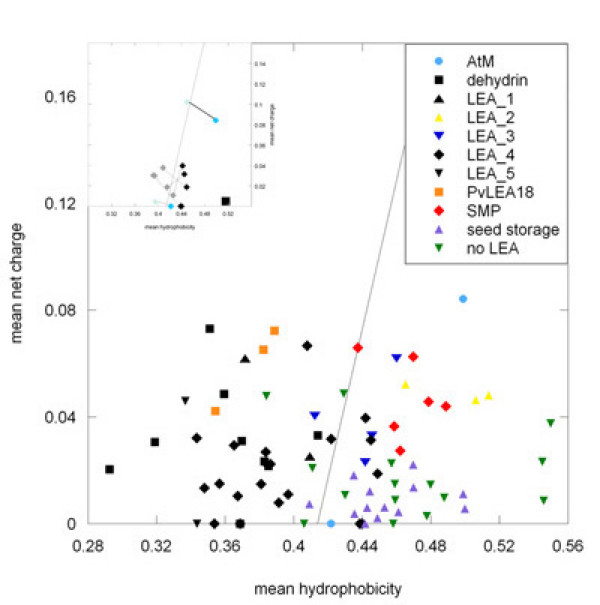
**Plot of mean net charge versus mean hydrophobicity of LEA and selected other proteins**. "No LEA" refers to proteins originally annotated as LEA proteins but re-annotated in our study (Additional file 1). Arabidopsis seed storage proteins were included in the analysis, because they are a group of seed proteins that have clearly no sequence similarities to LEA proteins. The line marks the border between natively unstructured (left) and folded (right) proteins [91]. The inset documents the shift of five proteins when the putative targeting sequence is removed.

As a first step towards this goal, we have used a simple computational prediction of the propensity of Arabidopsis LEA proteins to be natively unfolded. Figure 5 shows a plot of the mean net charge as a function of mean hydrophobicity of all investigated Arabidopsis proteins. The line marks the empirical border of natively unstructured and folded proteins [91]. This plot indicates that most of the LEA proteins are unstructured, whereas those proteins we annotated as not being LEAs (Additional file 1) together with seed storage proteins from Arabidopsis are mostly predicted to be folded. It is striking that all members of the LEA_2 group are predicted by this analysis to be folded. This is in agreement with the crystal structure determined from LEA14 [89], indicating that this is a general feature of LEA_2 group proteins. Also, the SMP group proteins are exclusively predicted to be folded, as are the AtM LEA proteins and a few members of the LEA_4 group. However, the LEA_4 group and AtM proteins contain putative targeting sequences which are known to be hydrophobic. When the targeting sequences were removed, all proteins shifted to a position indicating a lack of structure (Fig. 5, inset). This raises the question whether the folded proteins should really be called LEA proteins *sensu strictu*, or whether an unfolded structure in solution is a defining property of LEA proteins. A final answer to this question will have to await information about the functional significance of this property.

Computational prediction of the subcellular distribution of the LEA proteins using targetP indicates further differences between the LEA groups. Whereas the members of most groups are localized in the cytosol ("other" in Table 1 indicates that no signal peptide was detected), LEA_4 proteins are predicted to be present in all cellular compartments, the LEA_3 proteins are exclusively targeted to chloroplasts and mitochondria and the two AtM proteins are predicted to enter the secretory pathway. In the SMP group, one member (#49) is probably targeted to chloroplasts.

Experimental evidence for the subcellular localization of approximately 15 different LEA proteins has been published so far. Of the Arabidopsis LEA proteins, the cold induced LEA_4 group protein COR15A (#24) is localized in the chloroplast stroma [98], as is most likely the highly homologous COR15B (#23). In addition, the SMP group protein RAB28 (#31) is localized in the nucleus and it is likely that AtEPC31 (#32) has the same localization, because it contains the same targeting sequence [99]. No other data for Arabidopsis LEA proteins are available. The general conclusion from both prediction and published experimental evidence is that LEA proteins can be present in all subcellular compartments. Whether they have different functions in different compartments and what these functions are remains to be determined.

### Genomic organization of the Arabidopsis genes encoding LEA proteins

**Figure 6:**
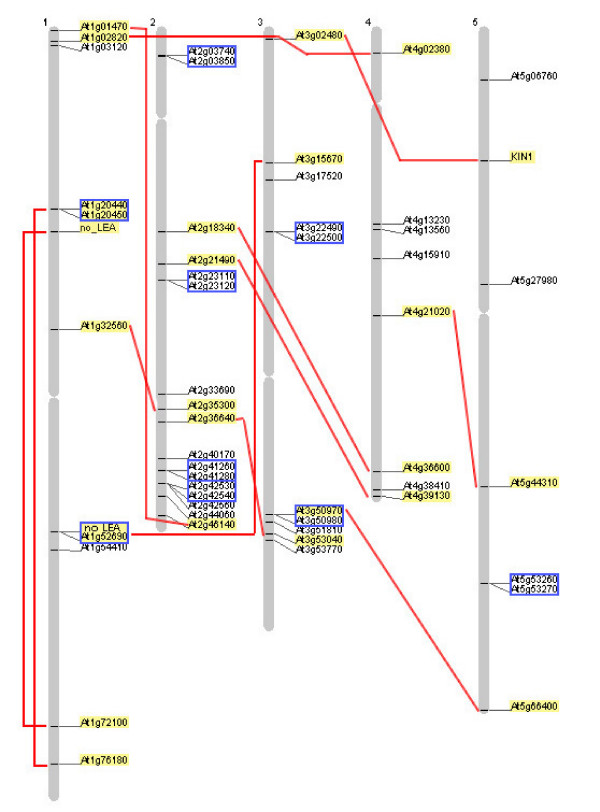
**Localization of the 51 identified *LEA *genes on the Arabidopsis chromosomes**. Genes related by endo-reduplication events during genome evolution (homeologous genes) are connected by lines and highlighted. Genes present as tandem repeats in the genome are boxed in.

A plot of the *LEA *genes on the Arabidopsis genome shows that *LEA *loci can be found on every chromosome (Fig. 6). However, the density of these loci is very high on the lower arm of chromosome 2, which contains 29% of all *LEA *genes. The lower arm of chromosome 3 also contains a region with a number of *LEA *loci as well as the upper arm of chromosome 1. Seventeen *LEA *genes (33%) are found in tandem repeats resulting from local duplications of small parts of a chromosome (Table 6). These include the previously reported cases of *COR47 *(#4) and *ERD10 *(#5) [67], *COR15B *(#23) and *COR15A *(#24) [66], AtM17 (#21) and AtM10 (#22) [57], *RAB28 *(#31) and *AtEPC31 *(#32) [100], and *XERO2 *(#33) and *XERO1 *(#34) [101], in addition to the uncharacterized gene pairs #11 and #12, #15 and #16, as well as #49 and #50. Gene #7 is part of a tandem repeat with a gene that has no corresponding LEA domain.

**Table 6:** Tandem repeats of *LEA *genes in the Arabidopsis genome

**Tandem repeats**									
						**BLAST 2 SEQUENCES alignment**			

**gene 1**	**gene 2**	**gene 1 (number)**	**gene 2 (number)**	**Cellular loca-lization gene1**	**Cellular loca-lization gene2**	**raw score**	**bit score**	**expectation value**	**gene correlator (R**^2^**)**

At1g20440	At1g20450	4	5	Cytosol	Cytosol	392	155	2.00E-36	0.723
At1g52680	At1g52690	no LEA	7	Cytosol	Cytosol	102	43.9	0.002	0
At2g03740	At2g03850	11	12	Chloroplast	Chloroplast	542	213	5.00E-54	0.893
At2g23110	At2g23120	15	16	Cytosol	Cytosol	194	79.3	4.00E-14	0.033
At2g41260	At2g41280	21	22	secreted	secreted	154	63.9	9.00E-09	0.45
At2g42530	At2g42540	23	24	Chloroplast	Chloroplast	464	183	2.00E-45	0.695
At3g22490	At3g22500	31	32	Cytosol	Cytosol	856	334	4.00E-90	0.857
At3g50970	At3g50980	33	34	Cytosol	Cytosol	144	60.1	7.00E-08	0.192
At5g53260	At5g53270	49	50	Chloroplast	Cytosol	646	253	4.00E-66	n/a*

Comparison of predicted subcellular localization (targetP) and expression (gene correlator on Genevestigator website) of tandem repeats of *LEA *genes. The coding sequences of the genes were aligned using BLAST 2 SEQUENCES to quantitate the sequence differences between the genes in a pair (gene 1 and gene 2). To test for diversification in expression patterns, the gene correlator function on the Genevestigator website was used to compare expression patterns from publicly available expression profiling data.

*this gene pair is not included on the Affymetrix ATH1 array and therefore no correlation value could be calculated.

It is widely accepted that the Arabidopsis genome is the result of ancient genome duplication events and a following loss of genes from the tetraploid genome that resulted in the current diploid genome (see [102,103] for recent reviews). Ten pairs of such homeologous *LEA *genes could be identified on different chromosomes using the Arabidopsis Syntenic Pairs/Annotation Viewer [104]. In addition, two pairs were identified that contain one *LEA *gene and one gene that was not classified as a *LEA *gene (Fig. 6, Table 7). This means that 22 of the 51 *LEA *genes (43%) are parts of homeologous pairs. It has been estimated that approximately 26% of all genes in the Arabidopsis genome belong to such pairs [105,106], indicating that the number of duplicated *LEA *genes retained in the genome is above average.

**Table 7:** Duplications of *LEA *genes in the Arabidopsis genome

**Duplications**									
						**BLAST 2 SEQUENCES alignment**			

**gene 1**	**gene 2**	**gene 1 (number)**	**gene 2 (number)**	**Cellular loca-lization gene1**	**Cellular loca-lization gene2**	**raw score**	**bit score**	**expectation value**	**gene correlator (R**^2^**)**

At1g01470	At2g46140	1	27	Cytosol	Cytosol	484	191	1.00E-47	0.018
At1g02820	At4g02380	2	38	Chloroplast	Chloroplast	251	101	1.00E-20	0.014
At1g20450	At1g76180	5	10	Cytosol	secreted	386	153	1.00E-35	0.429
At1g32560	At2g35300	6	18	Cytosol	Cytosol	216	87.8	1.00E-16	0.819
At1g52690	At3g15670	7	29	Cytosol	Cytosol	453	179	7.00E-44	0.82
At1g72100	At1g22600	9	no LEA	secreted	secreted	317	126	2.00E-27	0.908
At2g18340	At4g36600	13	43	secreted	Mitochondrion	483	190	7.00E-47	0.966
At2g21490	At4g39130	14	45	Cytosol	Cytosol	146	60.8	4.00E-08	0.772
At2g36640	At3g53040	19	36	Cytosol	Cytosol	446	176	1.00E-42	0.945
At3g02480	At5g15960	28	no LEA	Cytosol	Cytosol	97	42	8.00E-03	0.043
At3g50970	At5g66400	33	51	Cytosol	Cytosol	124	52.4	2.00E-05	0.239
At4g21020	At5g44310	42	48	Mitochondrion	Chloroplast	503	198	3.00E-49	0.872

Comparison of predicted subcellular localization (targetP) and expression (gene correlator on Genevestigator website) of homeologous pairs of *LEA *genes. The coding sequences of the genes were aligned using BLAST 2 SEQUENCES to quantitate the sequence differences between the genes in a pair (gene 1 and gene 2). To test for diversification in expression patterns, the gene correlator function on the Genevestigator website was used to compare expression patterns from publicly available expression profiling data.

After a polyploidy event, redundant duplicated genes will be lost from the genome due to random mutation and loss of function. The same can be assumed for genes duplicated in tandem repeats. It has been suggested that duplicated genes are mainly saved from removal through functional diversification, because of positive gene dosage effects, or because they are indispensible parts of a protein network, e.g. as subunits in an enzyme or signalling complex [102,106-110]. The latter factor can be largely excluded for LEA proteins, as no indication of any enzymatic function has ever been reported and they lack any domains that might link them to enzymatic or signalling complexes.

In most of the homeologous pairs and tandem repeats evidence for functional diversification could be found from either gene expression patterns, sequence divergence, or the predicted subcellular localization (compare Table 1). For the tandem repeats, *COR15B *(#23), for instance, was upregulated under cold and salinity stress, while *COR15A *(#24) was also induced by drought, as reported before [66]. Gene #15 and *XERO1 *(#34) transcripts were only detected in seeds, while transcripts from their respective partner genes #16 and *XERO2 *(#33) could be detected in seeds and in vegetative tissues before and after stress induction. The tandem gene of gene #7 contains no recognizable LEA_4 domain, indicating sequence divergence. In one case, the protein encoded by one gene in a tandem pair (#49) was predicted to be targeted to the chloroplasts, while the other (#50) showed no targeting signal. This prediction, however, obviously needs to be experimentally tested before any definite conclusions can be drawn.

In the homeologous pairs, the three that contain one *LEA *gene and one gene that does not belong to any *LEA *gene family (Table 7) are obvious examples of changes in the protein sequence after duplication. In the dehydrin group, eight out of ten genes are linked by duplication, either as tandem repeats or homeologous pairs. These eight genes fall into three sequence groups, containing *COR47/ERD10/ERD14*, *XERO1/XERO2/RAB18*, and #16/#47 (Fig. 3). A similar grouping has also been obtained from an unrooted phylogenetic tree ([53]; compare also Fig. 1), however, without any reference to the underlying gene duplication events. The three proteins in the first group show an almost identical segmental structure, with little indication of functional diversification. The three genes in the second group and the two in the third show a high degree of variability in their segmental content (Fig. 3), indicating possible functional divergence. Unfortunately, there is no information available about the functional consequences of these changes in amino acid sequence, but the present analysis clearly identifies interesting targets for future study.

A difference in the predicted subcellular localization of members of homeologous pairs (Table 7) was found in three cases (#42 and #48; #13 and #43; ERD10 (#5) and #10). However, none of these have been experimentally verified. To further test for diversification in expression patterns on a broader basis [105,107,109,110], we have used the "gene correlator" function on the Genevestigator [111] website, which compares expression patterns from publicly available expression profiling data (Table 6 and 7). This analysis shows that for both tandem repeats and homeologous pairs the correlation coefficient varies between r^2 ^= 0.014 and 0.945. Diversification of the expression pattern is especially striking in the pairs #2 and # 38 (*SAG21*), #28 and At5g15960 (*KIN1*; no LEA), and #1 (*LEA14*) and #27, with r^2 ^= 0.014, 0.043, and 0.018, respectively. Interestingly, while #28 and *KIN1 *show a very low level of correlation in expression, the other pair between a *LEA *and a non-*LEA *gene (#9 and At1g22600) shows a high correlation (r^2 ^= 0.908). Also the tandem repeat between a *LEA *and a non-*LEA *gene (#7 and At1g52680) shows a high diversification in expression (r^2 ^= 0). This suggests that changes in the coding sequence, as indicated by the *LEA*/non-*LEA *classification, and in the promoter sequence that determines the expression pattern may be independent of each other.

In summary, we have identified 12 homeologous pairs and nine tandem repeats among the 51 *LEA *genes in Arabidopsis. Ten of the 12 homeologous pairs and six of the nine tandem repeats showed clear evidence for functional diversification at the levels of coding sequence, subcellular localization, or expression pattern. For those genes that did not show diversification in our analysis, a final conclusion has to await the identification of the physiological and biochemical function of the proteins, as only this would enable us to judge whether small differences in the coding sequence may have effects on the functional properties of the proteins. This has recently been demonstrated for two highly similar LEA proteins from the rotifer *Adineta ricciae*, where one protein protects enzymes during drying, while the other shows membrane association in the dry state [16]. In addition, it remains possible that duplication of such genes leads to a larger or more rapid accumulation of functionally redundant proteins and that this may be a selective advantage for the organism under some environmental stress conditions.

## Conclusion

LEA proteins have been found in phylogenetically distant organisms and have always been related to abiotic stress tolerance, especially desiccation tolerance. However, no unifying concept for their physiological role(s) and modes of action has been attained so far. This is in part due to the fact that research has been fragmented between different species, different groups of LEA proteins and different experimental approaches. In this paper, we have presented a genome-wide survey of LEA proteins and genes in Arabidopsis. The experimental and *in silico *analyses indicate a wide range of sequence diversity, intracellular localizations, and expression patterns. The high fraction of retained duplicate genes and the inferred functional diversification indicate that they confer an evolutionary advantage for an organism under varying stressful environmental conditions. The future elucidation of the physiological roles of these proteins and the relationship of their structures, and especially their large structural flexibility, with their modes of action should greatly benefit from the presented comprehensive analysis.

## Methods

### Plant material and growth conditions

*Arabidopsis thaliana *(accession Col-0) was grown in soil in a greenhouse at 16 h day length with light supplementation to reach 200 μE m^-2 ^s^-1 ^and a temperature of 20°C during the day and 18°C during the night, as described before [112]. Six-week-old plants were sampled for adult rosette leaves and roots. Seedling samples were taken 14 days after sowing. Bud, flower and stem samples were taken from plants with a fully grown inflorescence (nine weeks after sowing), and green siliques were harvested as they appeared. Plants for seed production (accessions Col-0, Col-2, L*er*, C24, Nd, Rsch) were grown as described above. The mature seeds were harvested and stored for one year (12°C, 30% RH). For stress treatments, five to six-week-old plants were subjected to mild drought (no watering under the growth conditions described above, harvest one day after the first signs of wilting (relative water content of treated plants approximately 97% of control plants), high light (~400 μE m^-2 ^s^-1 ^for seven days) and cold (4°C for 14 days at 90 μE m^-2 ^s^-1^). Leaves infected with powdery mildew (*Erysiphe cichoracearum*) were harvested six weeks after sowing [113]. Salt stress was applied in hydroponic culture on modified Hoagland medium (1.5 mM CaNO_3_, 1.26 mM KNO_3_, 0.75 mM MgSO_4_, 0.5 mM KH_2_PO_4_, 100 μM H_3_BO_3_, 100 μM Na_2_SiO_3_, 70 μM Fe-EDTA, 50 μM KCl, 10 μM MnSO_4_, 2 μM ZnSO_4_, 1.5 μM CuSO_4_, 75 nM Na_2_MoO_4_; K. Köhl, unpublished). The medium was changed every week and after six weeks, 100 mM NaCl was added to the medium. Leaves were harvested after an additional seven days. Control samples were taken from plants grown under the same conditions, but without additional NaCl. Heat shock was applied to detached leaves for 2 h at 37°C as described previously [114].

Axenic cultures were grown in full-nutrition medium [115] for 7 d after imbibing sterilized seeds for 3 d at 4°C. Abscisic acid (ABA) or giberellic acid (GA3) were added to a final concentration of 10 mM. Cultures were harvested after 6 h of induction.

### Quantitative RT-PCR (qRT-PCR)

For the isolation of total RNA, tissue samples from 15 plants or axenic cultures from five different flasks were pooled and homogenized under liquid nitrogen. Total RNA was isolated either by the "hot borate" method [116] or using TRIZOL reagent (Invitrogen). Approximately 30 μg total RNA were treated with RNase-free DNase (Roche). The absence of genomic DNA in the samples was verified by qPCR using intron-specific primers (Additional file 6), the RNA concentration and quality were assessed by photometric measurement (Biophotometer, Eppendorf) and gel electrophoresis (2001 Bioanalyzer, RNA 6000 Nano Chip Kit, Agilent Technologies). Approximately 3 μg total RNA was utilized to synthesize single-stranded cDNA using reverse transcriptase (SuperscriptIII, Invitrogen) and oligo-dT_18 _primers, according to the manufacturer's instructions. The cDNA was diluted 20- to 40-fold.

QRT-PCR primers were designed using the PrimerExpress 2.0 software (Applied Biosystems), with an amplicon length range from 61 to 161 bp, yielding primers of 19 to 24 bp with a melting temperature of 58–62°C (see Additional file 6 for all primer sequences). PCR reactions were performed in optical 384-well plates using the ABI PRISM 7900 HT Sequence Detection System (Applied Biosystems, USA). The SYBR Green fluorescent dye was used to detect the synthesized dsDNA. A total reaction volume of 10 μl contained 5 μl 2× SYBR Green Master Mix Reagent (Applied Biosystems), 1 μl of diluted cDNA and 200 nM of each gene-specific primer. PCR conditions were those described in detail recently [117] and data were expressed as the cycle number necessary to reach a threshold fluorescence value (C_T_). The reported values are the means of two technical replica from one biological experiment.

Data were normalized to the mean C_T _of three reference genes (At4g27960, At5g46630, and At4g34270) that were found to be stably expressed across various tissues and conditions [118]. The mean PCR efficiency (E) for every primer pair was calculated using the linregPCR software [119]. The average C_T _values of two technical replica for each *LEA *gene and each reference gene was calculated and set to the power of the respective PCR efficiency (ΔC_T_). If the difference in the C_T _values between the two technical replica was above 1.5, the values were removed from the dataset. In addition, samples that had multiple peaks in the dissociation graph were dismissed because this indicates that the PCR reaction was unspecific. Relative gene expression values (compiled in Additional file 3) were calculated from these data as:

$$\text{relative gene expression}=\frac{\sum \Delta {\text{CT}}_{\text{Refgenes}}}{\Delta {\text{CT}}_{\text{Gene of interest}}•\text{Number of Refgenes}}$$

### *In silico *analysis of LEA genes

*LEA *genes in *A. thaliana *were identified by keyword search in Genbank, accessed through NCBI [120]. In addition, tblastn [121] searches were performed on the translated Arabidopsis genome with the protein sequences of the well-characterized *LEA *genes from *Gossypium hirsutum*.

The Pfam database of protein families and HMMs [122] was applied to characterize the proteins on the basis of their sequence homology to the stored Pfam domains [123]. In the Prosite [124,125] database, two patterns defining dehydrins are present. For most of the other groups, we were able to create patterns with the PRATT [126,127] tool. The PATTINPROT [128,129] tool was used to verify the stringency of the retrieved patterns by querying the UniProt [130,131] database. To get more information about the nature of the gene products, the GRAVY (grand average of hydropathy), the molecular weight and the pI were predicted by the PROTPARAM [132,133] tool. The signal peptide analysis was done by the TargetP [134,135] algorithm. The gene loci were plotted on the Arabidopsis chromosomes with the Chromosome Map Tool on the TAIR webpage [136]. Duplications were retrieved by the Arabidopsis Syntenic Pairs/Annotation Viewer [104]. The tandem repeats and homeologous pairs were aligned with the BLAST 2 SEQUENCES [137] tool on the NCBI webpage [120]. Multiple sequence alignments were performed using the ClustalW [138,139] algorithm and the unrooted dendrogram was drawn based on ClustaW alignments [140].

ABRE and LTRE *cis*-acting elements in the -2000 nt promoter region of the *LEA *genes were found by the plantPAG tool [141] querying the PLACE [77,142] database.

## Authors' contributions

MH carried out the experimental work and the in silico analyses, participated in the design of the study and in the data analysis and helped to draft the manuscript. DKH designed the study, participated in the data analysis and drafted the manuscript. Both authors read and approved the final manuscript.

## Supplementary Material

Additional file 1 **Wrongly annotated *LEA *genes in Arabidopsis**. Compilation of all genes that have been annotated as *LEA *genes in the Arabidopsis genome, but that we found not to be members of any LEA group. Click here for file

Additional file 2 **Appearance of segments in Arabidopsis dehydrins**. Classification of the dehydrins found in Arabidopsis according to the KYS and Lys-rich segment system. Click here for file

Additional file 3 **Expression data of all Arabidopsis *LEA *genes**. Quantitative Real-Time RT-PCR data for the expression of *LEA *genes in diverse Arabidopsis organs and under stress conditions and hormone treatments. Click here for file

Additional file 4 **ABRE and LTRE *cis*-elements in *LEA *gene promoters**. The table shows the number of the two core motifs found in the -2000 nt promoter regions of all 51 *LEA *genes. Click here for file

Additional file 5 **Details of the characteristics of all Arabidopsis LEA proteins**. Information on sequences and some predicted features of the 51 LEA genes. Click here for file

Additional file 6 **Sequences of all primers used in the quantitative RT-PCR experiments**. List of all primers used in the present study and their nucleotide sequences. Click here for file
